# Association between dexmedetomidine use for the treatment of alcohol withdrawal syndrome and intensive care unit length of stay

**DOI:** 10.1186/s40560-019-0405-1

**Published:** 2019-11-04

**Authors:** Ekaterina R. Yavarovich, Maythawee Bintvihok, Justin C. McCarty, Janis L. Breeze, Peter LaCamera

**Affiliations:** 1 Division of Pulmonary and Critical Care Medicine, Lahey Hospital and Medical Center, 41 Mall Road, Burlington, MA 01805 USA; 2 Department of Internal Medicine, St. Elizabeth’s Medical Center, Boston, MA USA; 3 Department of Surgery, St. Elizabeth’s Medical Center, Boston, MA USA; 4 Tufts Clinical and Translational Science Institute, Tufts University, and Institute for Clinical Research and Health Policy Studies, Tufts Medical Center, Boston, MA USA; 5 Division of Pulmonary, Critical Care Medicine and Sleep Medicine, St. Elizabeth’s Medical Center, Boston, MA USA

**Keywords:** Dexmedetomidine, Alcohol withdrawal, Benzodiazepine, Length of stay

## Abstract

**Purpose:**

Alcohol withdrawal syndrome (AWS) is commonly treated in medical ICUs and typically requires high resource utilization. Dexmedetomidine for AWS has not been extensively investigated, and guidelines regarding its use are lacking. We evaluated the association between dexmedetomidine use in AWS and ICU length of stay (LOS).

**Methods:**

We performed a multi-institutional retrospective cohort study of patients in the ICU with the primary diagnosis of AWS. ICU LOS of those treated with benzodiazepines alone vs. benzodiazepines plus dexmedetomidine was compared. Negative binomial regression was performed to test whether dexmedetomidine use was associated with increased ICU LOS after adjustment for age, gender, body mass index, and the time between hospital and ICU admission.

**Results:**

Four hundred thirty-eight patients from eight institutions were included. Patients treated with benzodiazepines plus dexmedetomidine had higher Clinical Institute Withdrawal Assessment for Alcohol scores at ICU admission, spent longer on the medical wards prior to ICU admission, and had longer unadjusted ICU LOS (*p* < 0.0001). After covariate adjustment, dexmedetomidine remained associated with longer ICU LOS (relative mean to non-dexmedetomidine group 2.14, 95% CI 1.78–2.57, *p* < 0.0001).

**Conclusions:**

Compared to benzodiazepines alone, dexmedetomidine for the treatment of AWS was associated with increased ICU LOS. These results provide evidence that dexmedetomidine may increase resource utilization.

## Introduction

Alcohol withdrawal syndrome (AWS) accounts for 9% of intensive care unit (ICU) admissions in the USA and 13% of overall ICU costs [1]. The contributors to these costs include the often prolonged ICU lengths of stay, staffing requirement related to behavioral challenges, and drug costs. AWS results from an imbalance between inhibitory and excitatory neurotransmitters resulting in profound agitation, autonomic hyperactivity, seizures, and delirium.

Benzodiazepines, γ-aminobutyric acid receptor (GABA) agonists, are considered first-line therapy in AWS as they are familiar medications with a long-standing record of efficacy. For these reasons, benzodiazepines are woven into most AWS treatment algorithms [2]. Severe AWS cases, however, can be resistant to benzodiazepines, and/or the necessary doses may lead to treatment-related adverse events such as respiratory depression. Clinicians, in these cases, look to alternatives or supplemental medications to benzodiazepines. Because the consequences of benzodiazepine-related respiratory depression can include a need for mechanical ventilation resulting in prolonged ICU length of stay (LOS), aspiration and/or ventilator-associated pneumonia, ICU-associated delirium, and increased healthcare costs, there has been interest in identifying treatments for AWS that control agitation and autonomic hyperactivity while avoiding respiratory depression [1, 3, 4].

Dexmedetomidine is an intravenous selective central alpha-2 receptor agonist which results in a cooperative sedation without depression of the respiratory drive. Currently, dexmedetomidine is approved by the FDA for procedural conscious sedation and sedation for mechanical ventilation less than 24 h. Additionally, it is often used off-label as an adjunct to benzodiazepines in clinical practice for treating severe AWS. Small studies have suggested that its use may lower the rate of intubation and allow for improved patient communication with care providers compared to benzodiazepines or propofol for severe AWS [5–7]. However, there is limited evidence supporting its use in AWS [4, 7, 8]. Published studies which include multiple, small retrospective series and two randomized trials suggest that adjunctive dexmedetomidine use in AWS is associated with a reduction in benzodiazepine dosing, possible avoidance of intubation, and rapid decreases in Clinical Institute Withdrawal Assessment for Alcohol (CIWA) scores. The therapy is limited in some patients by the cardiovascular side effects of bradycardia and hypotension. For this reason, dexmedetomidine infusions must be administered in an ICU setting. While there are recognized benefits and risks of using dexmedetomidine in AWS, the effect of the medication on ICU length of stay has not been well studied, and guidelines for its use are currently lacking.

In this context, we performed a retrospective, multi-institutional cohort study evaluating the association between adjunctive dexmedetomidine use for the initial treatment of AWS compared to benzodiazepine use alone and ICU length of stay. We hypothesized that dexmedetomidine use would be associated with longer ICU lengths of stay compared to benzodiazepines alone because of its need to be administered in an ICU setting.

## Methods

We conducted a multi-institutional retrospective cohort study of patients admitted to the ICU with the primary diagnosis of AWS within the Steward Health Care System in Massachusetts following the STROBE guidelines [9]. The study was approved by the St. Elizabeth’s Medical Center IRB (HW171). We compared patients treated with benzodiazepines (BZD group) alone to those treated initially with benzodiazepines plus dexmedetomidine (DEXBZD group). The primary outcome was the total ICU length of stay.

### Study cohort

For this study, eligible patients were defined as those admitted to the ICU at eight hospitals within the Steward Health Care System with a primary diagnosis of alcohol withdrawal. Cases were identified using the network’s eICU patient database. The study period for inclusion was January 1, 2012, to May 31, 2017. Exposure was defined as the administration of adjunctive dexmedetomidine within 1 h of arrival to the ICU for management of AWS. All subjects in this group also received benzodiazepines either prior to, during, or following dexmedetomidine infusion. The non-exposed group was managed with benzodiazepines alone. Exclusion criteria included any patient with an alternative primary indication for ICU admission other than alcohol withdrawal, intubation prior to or during an ICU admission, admission CIWA score of 0 or CIWA not recorded, patients who died during their ICU stay, patients transferred directly to the ICU from an outside hospital, patients who left the hospital against medical advice, and patients transferred to an alternative ICU. In the DEXBZD group, patients who either arrived to the ICU receiving dexmedetomidine or those started on dexmedetomidine within 1 h of arrival were included.

### Statistical analysis

Participant demographics were compared using two independent sample *t* tests for continuous data or chi-square test for categorical data. For our primary analysis, we compared ICU LOS between groups using negative binomial regression with generalized estimating equations to account for clustering of patients within hospitals. The adjusted model included age, gender, BMI, admission CIWA, and pre-ICU LOS. An exploratory interaction term between dexmedetomidine and admission CIWA was included to test for effect measure modification. We also performed stratified analyses by grouping patients based on their admission CIWA score into mild AWS (CIWA 1–10), moderate AWS (CIWA 11–20), and severe AWS (CIWA > 20), and evaluated the impact of dexmedetomidine on LOS within each stratum, adjusting for the same confounders as above. All statistical testing was two-sided with alpha = 0.05. SAS version 9.3 was used for the statistical analysis (SAS Institute, Cary, NC).

## Results

### Participant characteristics

**Fig. 1:**
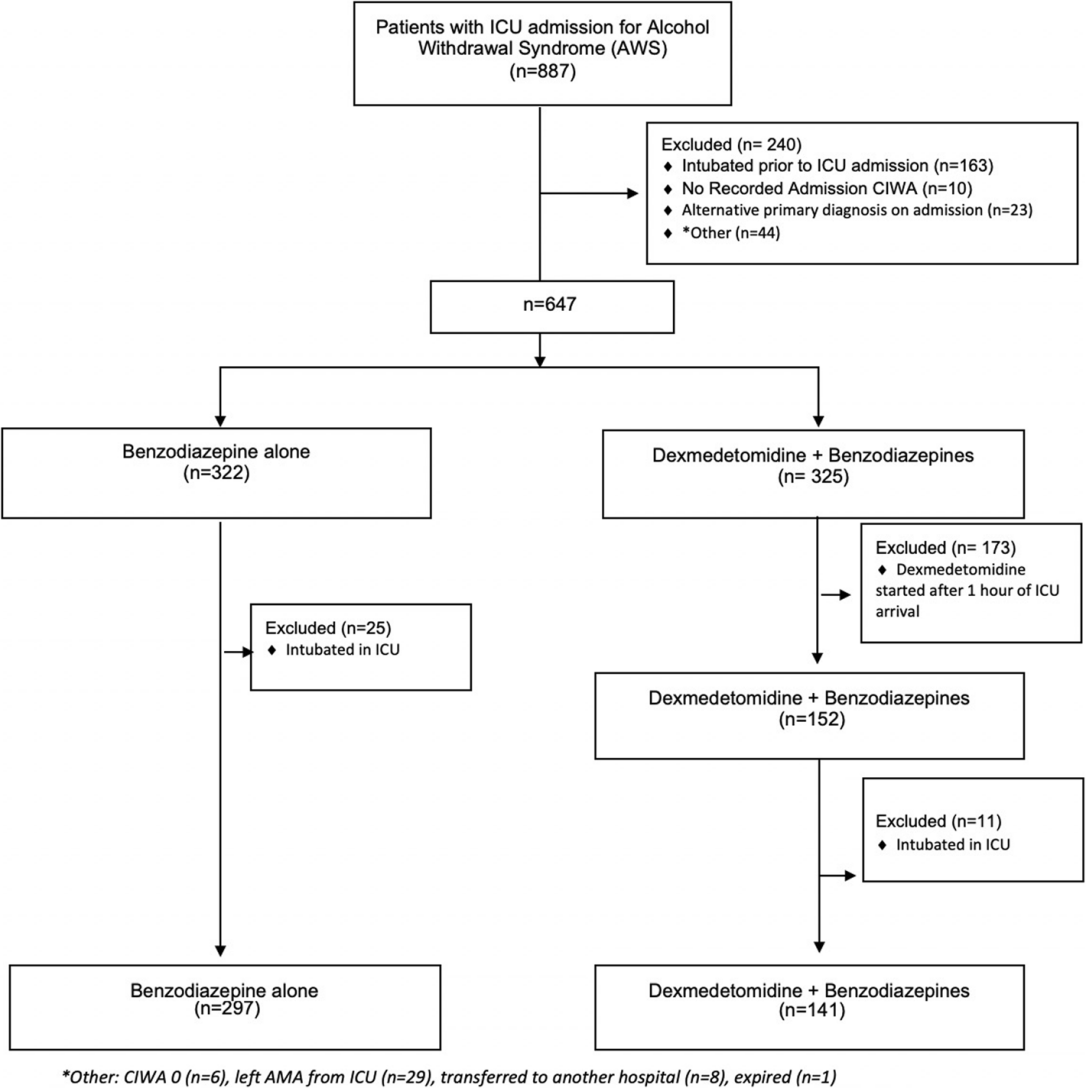
Flow diagram

Of the 887 alcohol withdrawal admissions reviewed, 438 met the study inclusion and exclusion criteria (Fig. 1). There was no difference in the proportion of patients who were excluded due to intubation following ICU admission between the BZD group and DEXBZD group (8.3% vs. 7.2%, *p* = 0.7). Among those included for analysis, 141 patients were included in the DEXBZD group and 297 in the BZD group. No patients were treated with dexmedetomidine alone, and none received phenobarbital. There were no significant differences in mean age and BMI between the groups; but the proportion of women in the DEXBZD group was significantly lower than the BZD group [16.3% vs. 25.2%, *p* = 0.03] (Table 1). Mean admission CIWA was higher in the DEXBZD group [mean (SD) 20.4 (10.2) vs. 15.5 (8.7), *p* < 0.0001]. The DEXBZD group had a longer pre-ICU LOS [mean (SD) 23.4 (32.4) h] than the BZD group [9.3 (18.3) h, *p* < 0.0001]. ICU discharge CIWA did not differ significantly between the two groups. The mean APACHE IVa scores between the DEXBZD [40.2 (13.0)] and BZD [39.7 (15.2)] were not significantly different (*p* = 0.7) overall as well as within the CIWA severity strata (*p* = 0.5) (Table 1).

**Table 1:** Patient characteristics

	Dexmedetomidine plus benzodiazepine, *n* = 141	Benzodiazepine alone, *n* = 297	*p* value
Female, *N* (%)	23 (16.3)	75 (25.2)	0.04
Age, mean (SD)	50.7 (11.3)	51.6 (12.5)	0.4
BMI, mean (SD)	25.4 (5.4)	25.9 (5.6)	0.4
Pre-ICU admission seizure, *N* (%)	12 (8.5)	33 (11.1)	0.4
Admission CIWA, mean (SD)	20.4 (10.2)	15.5 (8.7)	< 0.0001
Alcohol withdrawal severity, *N* (%)			< 0.0001
Mild (admission CIWA 1–10)	21 (14.9)	91 (30.6)	
Moderate (admission CIWA 11–20)	54 (38.3)	128 (43.1)	
Severe admission CIWA (> 20)	66 (46.8)	78 (26.3)	
APACHE IVa. mean (SD)	40.2 (13.0)	39.7 (15.2)	0.7
APACHE IVa within CIWA groups			
Mild (1–10)	39.5 (10.4)	38.9 (14.2)	0.5
Moderate (11–20)	38.5 (12.0)	40.3 (16.5)	
Severe (> 20)	41.8 (14.6)	39.6 (14.4)	
Pre-ICU LOS (h), mean (SD)	23.4 (32.4)	9.3 (18.3)	< 0.0001
ICU Discharge CIWA, mean (SD)	6.6 (4.7)	6.4 (5.2)	0.6
ICU LOS (h), mean (SD)	88.7 (74.0)	37.3 (36.2)	< 0.0001
Duration of dexmedetomidine treatment (h), mean (SD)	60.9 (53.7)	–	
Mild CIWA (1–10)	47.9 (41.1)		
Moderate CIWA (11–20)	80.5 (60.1)		
Severe CIWA (> 20)	48.9 (46.3)		

There was institutional variability in the adjunctive utilization of dexmedetomidine among the eight different hospitals with the highest utilizer administering dexmedetomidine in 57% of cases (*n* = 37) while the lowest utilizer administered dexmedetomidine in 14% of cases. No patients included in the analysis received phenobarbital or clonidine for the treatment of their alcohol withdrawal. Additionally, no patients were discharged from the ICU while receiving a dexmedetomidine or benzodiazepine infusion.

### Impact of dexmedetomidine on length of stay

In the unadjusted analysis, ICU length of stay was significantly higher in the DEXBZD group compared with the BZD group [88.7 vs. 36.3 h; *p* < 0.0001] (Table 1). After adjustment for age, gender, BMI, AWS severity, and pre-ICU LOS, the DEXBZD group had a longer ICU LOS (relative mean to the non-dexmedetomidine group 2.14, 95% confidence interval (CI) 1.78–2.57, *p* < 0.0001) (Table 2), corresponding to a difference of 42.9 h (95% CI 29.4–61.2). There was no significant interaction between the treatment group and AWS severity, and the term was not kept in the models.

**Table 2:** Unadjusted and adjusted comparison of ICU length of stay comparing dexmedetomidine use to benzodiazepine treatment alone

	Unadjusted (univariate) model			Adjusted model**		
	Ratio*	95% confidence interval	*p* value	Ratio*	95% confidence interval	*p* value
Dexmedetomidine plus benzodiazepine	2.18	1.83, 2.60	< 0.0001	2.14	1.78, 2.57	< 0.0001

*Relative mean number of ICU LOS hours: dexmedetomidine plus benzodiazepine to benzodiazepine alone group

**Adjusted for age, gender, BMI, alcohol withdrawal severity (based on admission CIWA), and pre-ICU length of stay

In the adjusted analyses stratified by admission CIWA (1–10, 11–20, > 20), the ICU LOS remained higher in the DEXBZD group within each stratum (Table 3).

**Table 3:** Stratified analyses for association between dexmedetomidine and ICU length of stay

Alcohol withdrawal severity	Unadjusted (univariate) models			Adjusted models**		
	Ratio*	95% confidence interval	*p* value	Ratio*	95% confidence interval	*p* value
Mild (admission CIWA 1–10) (*n* = 112)	1.99	1.37, 2.91	0.0003	2.23	1.58, 3.15	< 0.0001
Moderate (admission CIWA 11–20) (*n* = 182)	2.50	1.95, 3.21	< 0.0001	2.51	1.98, 3.18	< 0.0001
Severe (admission CIWA > 20) (*n* = 144)	1.88	1.45, 2.44	< 0.0001	1.99	1.44, 2.74	< 0.0001

*Relative mean number of ICU LOS hours: dexmedetomidine plus benzodiazepine to benzodiazepine alone group

**Adjusted for age, gender, BMI, and pre-ICU length of stay

## Discussion

Our study found that in a cohort of patients admitted to the medical ICU with alcohol withdrawal, initial treatment with dexmedetomidine in addition to benzodiazepines was associated with more than a twofold increase in ICU length of stay compared to benzodiazepine use alone.

The justification for dexmedetomidine use is based on its effect on alpha-2 receptors which reduces the hyper-adrenergic state in AWS thus suppressing tachycardia and anxiety while promoting arousable sedation [5, 7]. In comparison with clonidine, dexmedetomidine has a half-life of 2.3 h vs. 6 h and a higher selectivity for alpha-2 receptors [5]. Unlike benzodiazepines and phenobarbital, dexmedetomidine does not have GABA activity and therefore does not cause respiratory depression. Because of these properties, dexmedetomidine use may result in a decreased overall need for benzodiazepines and a reduction in intubation rates in AWS patients.

The finding of significantly longer length of stay for those treated with dexmedetomidine in our study corresponds to prior literature which showed that combination therapy with benzodiazepine and dexmedetomidine lengthened ICU LOS from 2.9 days compared to 1.4 days for benzodiazepine use alone [10]. This study had several limitations, however: a small sample size of 29 and 38 patients in each arm, inclusion of intubated patients who are commonly sedated with dexmedetomidine, a delay of > 24 h in starting dexmedetomidine in most patients, and the analysis did not control for AWS severity. A single prospective, randomized, double-blind, placebo-controlled dose range study of dexmedetomidine as an adjunct for alcohol withdrawal has been performed [7]. Mueller et al. randomized 24 patients to high dose vs. low dose dexmedetomidine or placebo and found that short-term dexmedetomidine had a benzodiazepine sparing effect [7, 10]. The ICU LOS of 5.5 days vs. 4 days was not statistically significant in that study comparing the low dose dexmedetomidine to benzodiazepines alone, though this study was underpowered for this outcome, while Beg et al. found in their retrospective cohort study of 77 patients a significant increase in ICU LOS of 2.9 days vs. 1.4 days in the dexmedetomidine group compared to the benzodiazepine alone [10].

Our study can be distinguished from previous reports through the larger number of patients included, the stratification of subjects according to admission CIWA, and the inclusion of subjects who were only treated initially with dexmedetomidine (upon arrival or within 1 h of admission to ICU) as opposed to categorizing patients in the dexmedetomidine group if there had been any exposure during an ICU stay. We did not focus upon or report the total doses of dexmedetomidine and/or benzodiazepines provided during ICU admissions which is potentially a limitation of the study. While medication doses may serve as a surrogate for alcohol withdrawal severity and outcome, we instead considered ICU admission and discharge CIWA scores and ICU length of stay to be more clinically meaningful measures of withdrawal outcomes. Integrating medication doses with additional clinical metrics is an important consideration for future studies.

Dexmedetomidine is typically considered an adjunctive therapy for the management of AWS used when primary medications are thought to be insufficient for symptom control. With an increase in the familiarity of dexmedetomidine in this clinical setting, its use appears to have risen. Multiple studies of patients with severe AWS who receive early, aggressive, intermittent, symptom-triggered doses of benzodiazepines have shown that an escalation of symptoms can be avoided when AWS is managed early and effectively [2, 11]. Increasingly, phenobarbital is also being used as the primary therapy for AWS [12]. The patients in our cohorts did not receive phenobarbital though this agent is now commonly used at our medical center and others involved in this study.

We speculate that there are at least two explanations to account for the association between dexmedetomidine use in AWS and prolonged ICU lengths of stay. Dexmedetomidine potentially suppresses AWS signs and symptoms without treating the underlying withdrawal physiology as it has no GABA modulation effects. This may alter the kinetics of withdrawal in a manner that potentially prolongs its duration. Additionally, as noted above, the infusion of dexmedetomidine requires ICU level of care because of its potential to cause cardiovascular complications. Those patients who received prolonged infusions of dexmedetomidine may have remained in the ICU simply because of the monitoring requirements of the medication.

The study findings must be viewed within the context of the study design. This is a retrospective cohort study, and thus, there is potential for unmeasured confounding and residual confounding within the adjusted variables. We accounted for confounding within our model by including only patients that were started on dexmedetomidine no later than 1 h following admission to the ICU and by stratifying patients by their CIWA scores. This was to attempt to control for treatment bias in patients in whom benzodiazepine therapy was deemed inadequate to control symptoms and also to group patients according to the stage or severity of their alcohol withdrawal process [5]. Furthermore, by accounting for clustering within the model by treatment facility, we adjusted for random effects from facility-level variation. We did not incorporate comorbidities in the model which could be viewed as potential confounders; however, AWS was considered the primary indication for ICU admission for all subjects as opposed to other common alcohol-related medical conditions such as pancreatitis, gastrointestinal bleeding, and acute hepatitis, and APCAHE IVa scores at ICU admission were similar between the two groups. Given the magnitude of impact observed upon LOS, it is unlikely that confounding would completely explain the effect seen. Furthermore, individual facility preference for giving dexmedetomidine is likely physician-driven preference rather than based on patient-level factors considering the disparate range of proportions of patients treated with adjunctive dexmedetomidine.

## Conclusion

The use of dexmedetomidine for the initial treatment of AWS in the ICU is associated with increased ICU LOS compared to the use of benzodiazepines alone. These findings were consistent across all strata of alcohol withdrawal severity. These results provide evidence that dexmedetomidine use is associated with increased resource utilization, and further studies are warranted to determine how best to incorporate dexmedetomidine into AWS treatment protocols.

## Data Availability

Data sharing is not applicable to this article as no datasets were generated or analyzed during the current study.

## Abbreviations

AWS: Alcohol withdrawal syndrome
BZD: Benzodiazepines
CIWA: Clinical Institute Withdrawal Assessment for Alcohol
DEXBZD: Benzodiazepines plus dexmedetomidine
